# Neurodevelopmental and psychiatric disorders in females with Turner syndrome: a population-based study

**DOI:** 10.1186/s11689-021-09399-6

**Published:** 2021-10-27

**Authors:** Hanna Björlin Avdic, Agnieszka Butwicka, Anna Nordenström, Catarina Almqvist, Agneta Nordenskjöld, Hedvig Engberg, Louise Frisén

**Affiliations:** 1 Centre for Psychiatry Research, Department of Clinical Neuroscience, Karolinska Institutet, & Stockholm Health Care Services, Region Stockholm, CAP Research Centre, Gävlegatan 22, SE-113 30 Stockholm, Sweden; 2 Department of Medical Epidemiology and Biostatistics, Karolinska Institutet, Stockholm, Sweden; 3 Department of Child Psychiatry, Medical University of Warsaw, Warsaw, Poland; 4 Child and Adolescent Psychiatry, Stockholm Health Care Services, Stockholm County Council, Stockholm, Sweden; 5 Department of Women’s and Children’s Health, Karolinska Institutet, Stockholm, Sweden; 6 Department of Pediatric Endocrinology, Astrid Lindgren Children’s Hospital, Karolinska University Hospital, Stockholm, Sweden; 7 Pediatric Allergy and Pulmonology Unit, Astrid Lindgren Children’s Hospital, Stockholm, Sweden; 8 Pediatric Surgery, Astrid Lindgren Children’s Hospital, Karolinska University Hospital, Stockholm, Sweden; 9 Department of Gynecology and Reproductive Medicine, Karolinska University Hospital, Stockholm, Sweden

**Keywords:** Turner syndrome, Psychiatric disorder, Neurodevelopmental disorder, ADHD, Autism spectrum disorder, Anxiety, Depression, Eating disorders, Substance misuse, Intellectual disability

## Abstract

**Background:**

Turner syndrome is the result of the partial or complete absence of an X chromosome in phenotypic girls. This can cause an array of medical and developmental difficulties. The intelligence quotient in females with Turner syndrome has previously been described as uneven, but considered within normal range. Although their social, intellectual, and psychiatric profile is described, it is unclear to what extent these females meet the clinical criteria for neurodevelopmental or psychiatric diagnoses. The aim of this study was to examine the prevalence of neurodevelopmental and psychiatric disorders in females with Turner syndrome.

**Methods:**

A retrospective cohort study was performed with a total of 1392 females with Turner syndrome identified through the Swedish National Patient Register and compared with 1:100 age- and sex-matched controls from the general population. The associations between Turner syndrome and diagnoses of neurodevelopmental and/or psychiatric disorders were calculated using conditional logistic regression and is presented as estimated risk (odds ratio, OR, 95% confidence interval, CI) in females with Turner syndrome compared with matched controls.

**Results:**

Females with Turner syndrome had a higher risk of neurodevelopmental or psychiatric disorder (OR 1.37, 95% CI 1.20–1.57), an eightfold increased risk of intellectual disability (OR 8.59, 95% CI 6.58–11.20), and a fourfold increased risk of autism spectrum disorder (OR 4.26, 95% CI 2.94‑6.18) compared with the controls. In addition, females with Turner syndrome had twice the risk of a diagnosis of schizophrenia and related disorders (OR 1.98, 95% CI 1.36–2.88), eating disorders (OR 2.03, 95% CI 1.42–2.91), and behavioral and emotional disorders with onset in childhood (OR 2.01, 95% CI 1.35–2.99).

**Conclusions:**

Females with Turner syndrome have an increased risk of receiving a diagnosis of neurodevelopmental or psychiatric disorder. This warrants extensive assessment of intellectual and cognitive functions from early age, and increased psychiatric vigilance should be a part of lifelong healthcare for females with Turner syndrome.

## Introduction

Turner syndrome (TS) is a genetic disorder associated with partial or complete monosomy of the X chromosome resulting in karyotype 45,X. TS is one of the most common sex chromosome abnormalities with an incidence ranging from 1/2000 to 1/2500 in live-born females [1, 2]. The complete or partial loss of one X chromosome in girls results in a variation of clinical findings that may include short stature, early loss of ovarian function, cardiac anomalies, hearing loss, visual impairment, and neurocognitive difficulties. The neuroanatomical structure and function of the brain are thought to be highly affected by the characteristics of the X chromosome. Despite a wide spectrum of variation within the group, the prevailing hypothesis is that the absence or mosaicism of one X chromosome often presents with a specific pattern of cognitive strengths and deficits [3–6].

Previous studies on relatively small clinical samples reported that most girls and women with TS have a full-scale intelligence quotient (FSIQ) within the normal range but with an uneven intellectual profile with different domains of relative strengths and deficits [7–12]. In addition, females with TS tend to have challenges in specific psychosocial and emotional areas such as behavioral skills, social awareness, and adaptation in social relationships [13–15]. Furthermore, they also have an increased risk of inattention and executive disabilities [16, 17], as well as impairments in face and emotion recognition, and direction of gaze [18–21]. While some studies show an association between TS and autism spectrum disorders (ASD) [22–24], other studies found that the preserved abilities and functions in individuals with TS, while reminiscent of ASD, do not show an increased risk of meeting the criteria for ASD [25, 26].

Studies on psychiatric disorders among women with TS are scarce [27, 28], but physical stigma, lack of peers, social difficulties, and low self-esteem might contribute to increased levels of depressive and anxiety symptoms, thus placing females with TS at risk of developing psychiatric morbidity [29, 30].

Given that previous studies are based on small, highly selected clinical samples often without population references, we carried out a nationwide retrospective cohort study to evaluate the risk of clinical diagnoses of psychiatric and neurodevelopmental disorders in individuals with TS in comparison with matched reference individuals from the general population. The secondary aim was to add to the understanding of the X chromosome and the etiology of neurodevelopmental and psychiatric disorders by gaining new knowledge of the clinical diagnoses associated with TS.

## Methods

### National registers

All citizens in Sweden have a unique personal identification number that is used in all official records and that enables linkage between population-based registers. For research purposes, Statistics Sweden has generated a key that converts the personal identification number to a unique ID number that results in pseudonymized, de-identified data for each individual. The key is not available to researchers [31]. We performed a matched cohort study using data from the National Patient Register (NPR) and the Total Population Register (TPR) [32]. In 1964, NPR began collecting information on Swedish inpatient care at public hospitals in a few Swedish counties; in 1973, discharge diagnoses for all psychiatric inpatients episodes were added; in 1987, the register included full nationwide coverage of inpatient diagnoses; and since 2001, the register includes all diagnoses from psychiatric outpatient care [33]. The Total Population Register (TPR) records data including birth, death, name change, marital status, family relationships, and migration status updated by the Swedish Tax Agency [32].

### Study design and participants

Females with TS were identified from NPR, both inpatient and outpatient healthcare, using the following diagnostic International Classification of Diseases (ICD) codes [34]: ICD-8 759.50, 310.54, 311.54, 312.54, 314.54, 315.54; ICD-9 758G; and ICD-10 Q96 [33]. Each individual with TS was matched with 100 unaffected female controls by county and date of birth from TPR. The risk of lifetime psychiatric outcomes was stratified depending on what year the individual was diagnosed with TS: (1) individuals diagnosed before 1987, (2) individuals diagnosed from January 1, 1987, through December 31, 2001, and (3) individuals diagnosed from January 1, 2002, through December 31, 2013. This stratification was chosen because it coincides with changes in NPR data collection: the NPR has full nationwide coverage of inpatient diagnoses since 1987 and full coverage of all diagnoses from psychiatric outpatient care since 2002. The study was approved by the Regional Ethical Review Board in Stockholm 2013/862-31.

### Outcomes

Psychiatric disorder was defined as any diagnosis within ICD-8 codes 290–315, ICD-9 codes 290–319, and ICD-10 codes F10–F99 in the NPR. The outcome measures were as follows: (1) any NDDs/psychiatric disorders, (2) mental and behavioral disorders due to psychoactive substance use, (3) schizophrenia and related disorders (including schizophrenia schizoaffective disorder), (4) mood disorders (including bipolar, single, and recurrent depressive disorder), (5) suicide attempts, (6) anxiety disorders (including dissociative, stress-related, and somatoform disorders), (7) eating disorders, (8) disorders of adult personality and behavior, (9) mental retardation (intellectual disability (ID)), (10) pervasive developmental disorders (autism spectrum disorders (ASD)), (11) hyperkinetic disorders (attention deficit hyperactivity disorder (ADHD)), and (12) behavioral and emotional disorders with onset in childhood. Information of diagnoses of neurodevelopmental and psychiatric disorders was drawn from NPR using the ICD codes. ICD-based definitions are shown in Table 1.

**Table 1:** ICD codes used to define neurodevelopmental and psychiatric disorders [34]

Diagnoses	ICD-10 codes, > 2001	Equivalent ICD-9 codes, 1987‑2001	Equivalent ICD-8 codes, < 1987
*Any neurodevelopmental disorders/psychiatric disorders*	F10-F99	290–319	290–315
*Mental and behavioral disorders due to psychoactive substance use*	F10-F19	291, 303, 304	291, 303, 304, 305A, 305X
*Schizophrenia and related disorders (including schizophrenia schizoaffective disorder)*	F20-29	295, 297, 298	295, 297–299
*Mood disorders (including bipolar, single, and recurrent depressive disorder)*	F30-39	296, 300E, 311	296, 300.40
*Suicide attempts*	X60-X84	E950-E959	E950-E959
*Anxiety disorders (including dissociative, stress-related and somatoform disorders)*	F40-48	300, 300A-300D, 300F–300X, 308-309	300.00–300.30,300.50–300.99, 307
*Eating disorders*	F50	307B, 307F	-
*Disorders of adult personality and behavior*	F60-F62, F69	301	301
*Mental retardation (ID)*	F70-F79	317–319	310–315
*Pervasive developmental disorders (ASD)*	F84	299A	-
*Hyperkinetic disorders (ADHD)*	F90	314	-
*Behavioral and emotional disorders with onset in childhood*	F91-98	312–313	-

*ID* intellectual disability, *ASD* autism spectrum disorder, *ADHD* attention deficit hyperactivity disorder

### Statistical analyses

Similar to previous studies [35], we performed a matched cohort study to evaluate the risk of neurodevelopmental and psychiatric disorders in individuals with TS. The condition logistic regression (PROC LOGISTIC with STRATA statement) was used to estimate the odds ratios (OR) with 95% confidence intervals (CI) for each outcome. Each individual with TS was clustered with her reference individuals matched by the year and county of birth. The statistical analyses were all conducted with the SAS software (version 9.3; Cary, NC, USA).

## Results

We identified 1392 females registered with TS between 1969 and 2013. In 1969, twelve individuals were registered in NPR with a diagnosis of TS. The oldest individual with TS found in NPR was born 1898 and the diagnosis was registered in 1975.

Analysis of the entire cohort (*n* = 1392) showed that individuals with TS had an eightfold increased risk of intellectual disability diagnosis (OR 8.59, 95% CI 6.58–11.20) and a fourfold increased risk of being diagnosed with ASD (OR 4.26, 95% CI 2.94–6.18) compared with unaffected controls (Table 2). Individuals with TS also had twice the risk of being diagnosed with schizophrenia and related disorders (OR 1.98, 95% CI 1.36–2.88), eating disorders (OR 2.03, 95% CI 1.42–2.91), or behavioral and emotional disorders with onset in childhood (OR 2.01, 95% CI 1.35–2.99). However, the likelihood of diagnosis of mental and behavioral disorders due to psychoactive substance use (OR 0.64, 95% CI 0.44–0.92) was lower in females with TS compared to unaffected controls. There was no significant association between TS and ADHD, anxiety disorders, suicide attempts, mood disorders, or disorders of adult personality and behavior (Table 3).

**Table 2:** Neurodevelopmental/psychiatric disorders in girls and women with Turner syndrome compared with age-matched controls (1:100 controls)

Diagnoses	Turner syndrome ***N*** (%)	Matched controls ***N*** (%)	OR (95% CI)	***P***
*Any neurodevelopmental disorders/psychiatric disorders*	283 (20.33)	21948 (15.77)	1.37 (1.20–1.57)	**< .0001**
*Mental and behavioral disorders due to psychoactive substance use*	30 (2.16)	4635 (3.33)	0.64 (0.44–0.92)	**0.0091**
*Schizophrenia and related disorders (including schizophrenia schizoaffective disorder)*	29 (2.08)	1493 (1.07)	1.98 (1.36–2.88)	**0.0012**
*Mood disorders (including bipolar, single, and recurrent depressive disorder)*	79 (5.68)	8606 (6.18)	0.91 (0.72–1.15)	0.4252
*Suicide attempt*	19 (1.36)	2158 (1.55)	0.88 (0.56–1.39)	0.5682
*Anxiety disorders (including dissociative, stress-related, and somatoform disorders)*	96 (6.90)	11210 (8.05)	0.84 (0.68–1.04)	0.1034
*Eating disorders*	32 (2.30)	1609 (1.16)	2.03 (1.42–2.91)	**0.0004**
*Disorders of adult personality and behavior*	17 (1.22)	1720 (1.24)	0.99 (0.61–1.60)	0.9613
*Mental retardation (ID)*	62 (4.45)	760 (0.55)	8.59 (6.58–11.20)	**< .0001**
*Pervasive developmental disorders (ASD)*	30 (2.16)	726 (0.52)	4.26 (2.94–6.18)	**< .0001**
*Hyperkinetic disorders (ADHD)*	22 (1.58)	1732 (1.24)	1.28 (0.83–1.96)	0.2771
Behavioral and emotional disorders with onset in childhood	26 (1.87)	1319 (0.95)	2.01 (1.35–2.99)	**0.0017**

*OR* odds ratio, *ID* intellectual disability, *ASD* autism spectrum disorder, *ADHD* attention deficit hyperactivity disorder

**Table 3:** Life-time neurodevelopmental/psychiatric disorders in females with TS stratified by year of Turner diagnosis and compared with age-matched controls (1:100 controls)

Diagnoses	< 1987				1987–2001				> 2001			
	Turner syndrome, ***N*** (%)	Matched controls, ***N*** (%)	OR (95%CI)	***P*** value	Turner syndrome, ***N*** (%)	Matched controls, ***N*** (%)	OR (95%CI)	***P*** value	Turner syndrome, ***N*** (%)	Matched controls, ***N*** (%)	OR (95%CI)	***P*** value
*Any NDDs psychiatric disorders*	81 (24.70)	5641 (17.20)	1.59 (1.23–2.04)	**0.0006**	94 (19.75)	8550 (17.96)	1.12 (0.90–1.41)	0.3168	108 (18.37)	7757 (13.19)	1.51 (1.21–1.87)	**0.0003**
*Mental and behavioral disorders due to psychoactive substance use*	9 (2.74)	1177 (3.59)	0.76 (0.39–1.47)	0.3927	9 (1.89)	1983 (4.17)	0.44 (0.23–0.86)	**0.0056**	12 (2.04)	1475 (2.51)	0.81 (0.45–1.44)	0**.**4529
*Schizophrenia and related disorders*	15 (4.57)	595 (1.81)	2.61 (1.54–4.42)	**0.0017**	7 (1.47)	497 (1.04)	1.42 (0.67–3.03)	0**.**3886	7 (1.19)	401 (0.68)	1.77 (0.83–3.77)	0**.**1759
*Mood disorders (including bipolar, single, and recurrent depressive disorder)*	25 (7.62)	2382 (7.26)	1.05 (0.70–1.59)	0.8039	22 (4.62)	3480 (7.31)	0.61 (0.40–0.94)	**0.0164**	32 (5.44)	2744 (4.67)	1.18 (0.82–1.70)	0**.**3808
*Suicide attempt*	5 (1.52)	440 (1.34)	1.14 (0.47–2.78)	0.7786	8 (1.68)	981 (2.06)	0.81 (0.40–1.64)	0**.**5473	6 (1.02)	737 (1.25)	0.81 (0.36–1.82)	0**.**5999
*Anxiety disorders (including dissociative, stress-related, and somatoform disorders)*	34 (10.37)	2915 (8.89)	1.19 (0.83–1.70)	0.3587	28 (5.88)	4555 (9.57)	0.59 (0.40–0.86)	**0.0035**	34 (5.78)	3740 (6.36)	0.90 (0.63–1.28)	0**.**556
*Eating disorders*	4 (1.22)	207 (0.63)	1.95 (0.72–5.32)	0.2344	19 (3.99)	793 (1.67)	2.48 (1.55–3.97)	**0.0008**	9 (1.53)	609 (1.04)	1.49 (0.77–2.92)	0**.**2679
*Disorders of adult personality and behavior*	6 (1.83)	509 (1.55)	1.18 (0.52–2.67)	0.6938	5 (1.05)	745 (1.57)	0.67 (0.28–1.62)	0**.**3376	6 (1.02)	466 (0.79)	1.29 (0.57–2.92)	0**.**5514
*Mental retardation (ID)*	14 (4.27)	123 (0.38)	12.0 (6.78–21.07)	**< .0001**	20 (4.20)	287 (0.60)	7.29 (4.58–11.59)	**< .0001**	28 (4.76)	350 (0.60)	8.45 (5.69–12.57)	**< .0001**
*Pervasive developmental disorders (ASD)*	7 (2.13)	84 (0.26)	8.56 (3.92–18.71)	**< .0001**	11 (2.31)	303 (0.64)	3.74 (2.02–6.91)	**0.0004**	12 (2.04)	339 (0.58)	3.63 (2.02–6.52)	**0.0003**
*Hyperkinetic disorders (ADHD)*	0 (0.00)	205 (0.63)	0.00 (0.00-)	**0.0428**	9 (1.89)	709 (1.49)	1.28 (0.66–2.49)	0**.**4882	13 (2.21)	818 (1.39)	1.62 (0.92–2.83)	0**.**1173
*Behavioral and emotional disorders with onset in childhood*	2 (0.61)	92 (0.28)	2.19 (0.53–8.99)	0.3316	6 (1.26)	424 (0.89)	1.43 (0.63–3.22)	0**.**4195	18 (3.06)	803 (1.37)	2.31 (1.43–3.74)	**0.0023**

*OR* odds ratio, *NDD* neurodevelopmental disorder, *ID* intellectual disability, *ASD* autism spectrum disorder, *ADHD* attention deficit hyperactivity disorder

*Due to difference in the observation time, percentage of life-time psychiatric outcome may not be comparable between cohorts

When stratifying the results by the year the individual received her TS diagnosis in the NPR, there was a significantly higher risk of having a diagnosis of psychiatric or neurodevelopmental disorder in the groups diagnosed with TS before 1987, OR 1.59 (1.23–2.04), and after 2002, OR 1.51 (1.21–1.87), compared with controls (Table 3). In contrast, the group diagnosed with TS between 1987 and 2002 showed a non-significant increase compared to controls, OR 1.12 (0.90–1.41).

Sub-analysis for specific diagnoses showed a significantly increased risk of ASD and ID in all three cohorts. Specifically, the cohort diagnosed with TS before 1987 had the highest risk of being diagnosed with ASD or ID compared to the controls. In addition, the cohort with a diagnosis of TS before 1987 was the only group that had a significantly increased risk of schizophrenia and related disorders and ADHD. The cohort diagnosed with TS between 1987 and 2001 showed a significantly increased risk of eating disorders compared to the other cohorts, as well as significant decreased risk of mood, anxiety, and mental and behavioral disorders due to psychoactive substance use. The cohort diagnosed with TS after 2002 was the only group that showed increased risk of behavioral and emotional disorders with onset in childhood, such as conduct or emotional disorders.

## Discussion

In this population-based study of Turner syndrome, we compared the risk of neurodevelopmental and psychiatric diagnoses in girls and women diagnosed with TS with matched reference individuals from the general population. Our study identifies a significantly increased risk of ID and other clinical diagnoses, such as schizophrenia and related disorders, and eating, behavioral, and emotional disorders with onset in childhood. The findings also confirm previous studies that have shown a high risk of ASD in females with TS [22–24]. This is also the first study to report on mental and behavioral disorders due to psychoactive substance use in individuals with TS, showing that girls and women with TS have a lower risk of diagnosis with mental and behavioral disorders due to psychoactive substance use compared to the general population.

The most important finding was more than eightfold increased risk of ID. The intellectual level in TS is described as slightly lower than average but within normal range [6–8]. However, with small numbers (*n* = 50) of TS individuals, previous studies have been largely underpowered to detect any difference in fulfilling the diagnostic criteria for ID [6, 8]. A higher incidence of ASD has previously been shown by Creswell and Skuse [22]. However, this is the first study to estimate risk in comparison with unaffected population controls. A contributing factor in identifying the high risk of ID and ASD in females with TS in Sweden is probably the almost complete coverage of the national patient registers, including individuals with severe intellectual disabilities or restrictive autistic behaviors that might hinder study participation. However, the increased incidence of ID and ASD might also reflect the fact that individuals presenting with major intellectual disabilities or restrictive autistic behaviors are more likely to be genetically investigated, and thus diagnosed with TS.

The increased risk of ASD in this study is consistent with previous findings that approximately 3% of individuals with TS fulfill the ICD-10 diagnostic criteria for autism [22], twice the estimated incidence in the general population (1.5%) [36]. These results reflect previously well-described social difficulties in individuals with TS, who in comparison with girls and women in the same age group seem to have fewer friends and engage in fewer social activities [14]. Increased interest in the social deficits in TS might implicate an increased awareness of ASD or autistic traits associated with TS. Nevertheless, large studies that present prevalence, based on diagnostic criteria, are lacking.

Stratifying the results in relation to time of diagnosis provides an interesting opportunity to spot possible differences between the time periods. However, the relatively small sample sizes lead to fewer opportunities to draw reliable conclusions. Individuals with TS who were diagnosed before 1987 (23.6%) had an increased risk of receiving a diagnosis of ASD or ID compared to the groups diagnosed in 1987–2002, or after 2002. This might, again, reflect that the patient register only covered discharge diagnoses before 1987, thus including only the women with the most pronounced phenotypes and disabilities in need of inpatient care. In addition, the diagnosis and traits of autism have gained more attention during the last decade, with changes in the reporting practices and diagnostic criteria, which also might reflect an increase in the number of individuals diagnosed with ASD in general [37, 38].

Previous studies have shown a clear association between TS and executive dysfunctions in TS described by the diagnostic criteria for ADHD [10, 39], but surprisingly few individuals in this study had been diagnosed with ADHD. Thus, women with TS may very well be under-diagnosed with ADHD, as these dysfunctions may have been attributed to the diagnosis of TS and not assessed as a separate dysfunction. Our results may primarily reflect a previous lack of knowledge about ADHD; however, it is important to stress the correct assessment of symptoms regardless of a present syndrome.

Literature is scarce regarding the risk of schizophrenia and related disorders, but one previous study found schizophrenia to have an incidence of 1% in a group of 325 individuals with TS [28]. However, women with a diagnosis of schizophrenia showed a threefold increase for a diagnosis of TS [40, 41]. Interestingly, the mosaic karyotype 45,X/46,XX was clearly overrepresented in women with schizophrenia compared to other karyotypes of TS [40, 41]. In comparison, in this study, the prevalence was 2.08% and the odds ratio 1.98 (95% CI 1.36–2.88), but included all schizophrenia and related disorders, not just schizophrenia. Solely evaluation of the diagnosis of schizophrenia alone can give misleading results, as the diagnosis of schizophrenia is not often confirmed in the first episode of psychosis, especially in adolescents [42].

Furthermore, our results show that girls and women with TS have twice the risk of receiving an eating-disorder diagnosis compared with unaffected controls (OR 2.03, 95% CI 1.42–2.91). There are few studies on TS and eating disorders, but in one study of 100 females with TS, 46 of them had previous psychiatric diagnoses, which of six individuals (13%) had been diagnosed with an eating disorders (five with anorexia and one with bulimia) [27]. In a European cross-sectional study of the self-reported data of 325 individuals with TS, 10.8% reported eating disorder as a previous or current psychiatric diagnosis [28]. When stratifying our results, women with a diagnosis of TS registered between 1987 and 2001 had a significantly higher risk of being diagnosed with eating disorders, which is consistent with the overall prevalence of anorexia nervosa among Swedish female twins, 1.20% [43]. This might be explained by increases in the rates of bulimia in the general population during the 1980s and early 1990s; since then, rates have remained constant or decreased slightly [44]. The higher risk of eating disorder in TS may also be a result of ascertainable bias, since women with TS have regular medical examinations and are thus diagnosed to a greater extent.

The risk of anxiety or mood disorders in individuals with TS in this study was not significantly greater than in the general population. In contrast, a previous study found that 52% of a sample of 100 females with TS met criteria for current or past depressive or anxiety disorders during their lifetime [27]. This can suggest that mood and anxiety disorders in TS remain largely undiagnosed, but a probable causal factor may also be that the data source in the current study, the NPR, does not include diagnoses from the primary care facilities where mild forms of anxiety and depression are treated. In our results, the frequency of mood- and anxiety-related diagnoses were lower overall in females with TS compared to controls, with the lowest numbers among women whose TS diagnosis was registered 1987–2001. However, this may also reflect a tendency to describe psychiatric symptoms and conditions in individuals with TS as sub-phenomena of the syndrome, rather than separate diagnoses.

The cohort of individuals diagnosed with TS after 2002 was the only cohort with a significant increase in risk of behavioral and emotional disorders with onset in childhood. Individuals in this cohort can be assumed to be younger compared to the other cohorts, as methods for recognizing and detecting genetic syndrome are likely to be more effective over time, increasing the likelihood of obtaining a diagnosis at a younger age. Since an ICD equivalent to the diagnostic group “behavioral and emotional disorders with onset in childhood” did not exist until 1987, and includes diagnoses preferably made in childhood, this excludes a large part of the study population who were children before 1987. Thus, it is not surprising that the group diagnosed 2002–2013 has a significantly higher risk, but no definite conclusions can be drawn from this difference, since cohorts are stratified by year of registered TS diagnosis, rather than year of birth.

Correct investigation and diagnosis are essential in order to access proper care [30] as well as make adjustments in school or at work, training interventions, and drug treatment [7, 45–47]. Early detection of sex chromosomal abnormalities and intervention has, in general, been shown to have a positive impact on psychosocial, cognitive, and reproductive ability [30].

Our results support previous molecular findings suggesting an effect of X-linked genetic information in the etiology of neurodevelopmental disorders [48, 49]. Further describing the phenotype in TS may also provide better understanding of sexual dimorphism, as well as clues to a deeper understanding of X-linked neurodevelopmental disorders [48, 50].

## Strengths and limitations

The strength of this study is the population-based design data from national registries with nationwide coverage. However, there are limitations related to register-based methodology. For example, the exact karyotype, whether it is mosaicism or not, cannot be distinguished by ICD codes in the registers. We stratified the results to examine whether they differed between the groups due to the tendency to identify different diagnoses of neurodevelopmental disorders and psychiatric disorders over time. However, no reliable results could be found, as the groups became too small and the coverage in the registers varied too much. In addition, all data is decoded, so we cannot link individuals with their medical history and can therefore not know at what exact age the individuals received their diagnosis, only when it was added to the register. Aspects of medical treatment cannot be assessed in this study since the Prescribed Drug Register has only been in place since 2005 [51], and thus would not mirror treatment in our cohort. Furthermore, girls and women with TS are under regular care and make frequent visits to health care, receiving more intense surveillance for psychiatric outcomes than the general population, thus increasing the possibility of ascertainment bias and Berkson’s bias. Health care is publicly funded with universal access to both primary care and non-primary care in Sweden [52]. However, primary care is not included by NPR and is thus not included in our data. The absence of data from primary care may explain an unexpectedly low risk of anxiety and depressive disorders since milder forms of anxiety and depression are treated in primary care. On the other hand, this reinforces indications that ADHD might be under-diagnosed in individuals with TS since ADHD is treated in non-primary care. Most diagnoses in NPR have a positive predictive value (PPV) of 85–95%, but the figures vary according to diagnosis [33]. Among psychiatric diagnoses, PPV have ranged from 86 to 95% for schizophrenia and related disorders [53, 54], and 96% for ASD [55]. However, the results only reflect diagnoses, and as always in registry studies, the diagnosis cannot be verified by going back to the patient’s chart.

## Conclusion

This study not only confirms prior findings regarding the phenotype of TS but also adds new knowledge by presenting higher prevalence of ID and ASD than previously reported. These findings indicate that assessments related to development, intellectual functions, and psychiatric symptoms are warranted from an early age in females with TS in order to offer proper treatment, training interventions, and adjustments in their everyday life.

## Data Availability

The datasets used and/or analyzed during the current study are available from the corresponding author on reasonable request.

## Abbreviations

ICD: International Classification of Diseases
NPR: National Patient Register
TPR: Total Population Register
CDR: Cause of Death Register
NND: Neurodevelopmental disorders
ADHD: Attention-deficit hyperactivity disorder
ASD: Autism spectrum disorders
